# Removal of the commercial reactive dye Procion Blue MX-7RX from real textile wastewater using the synthesized Fe_2_O_3_ nanoparticles at different particle sizes as a source of Fenton's reagent

**DOI:** 10.1039/c8na00129d

**Published:** 2019-01-02

**Authors:** Maha A. Tony, Shehab A. Mansour

**Affiliations:** Advanced Materials/Solar Energy and Environmental Sustainability (AMSEES) Laboratory, Basic Engineering Science Department, Faculty of Engineering, Menoufia University Shebin El-Kom Egypt dr.maha.tony@gmail.com +20 482235695 +20 482221549

## Abstract

The aim of the present study was to signify the role of the particle size of an iron source in the photo-Fenton system for textile dyeing wastewater oxidation. In this respect, a facile synthesis of Fe_2_O_3_ nanoparticles (Fe_2_O_3_ NPs) *via* a simple sol–gel route using FeCl_3_ with different molarities was investigated. The XRD patterns confirmed the formation of Fe_2_O_3_ nanoparticles. Furthermore, different molar concentrations of the FeCl_3_ precursor significantly influenced the size and shape of the nanoparticles. The treatment of wastewater effluents containing the reactive dye Procion Blue MX-7RX from a real textile dyeing facility, was investigated using the synthesized Fe_2_O_3_ NPs as a source of the Fenton's reagent photocatalyst. The reaction was initiated and enhanced using an artificial UV source for increasing the ˙OH radical yield. System parameters such as the initial dye load in wastewater, H_2_O_2_ and Fe_2_O_3_ NP concentrations, pH and the working temperature were investigated for process optimization. The quality of water in this investigation was examined by making measurements of chemical oxygen demand (COD), total suspended solids (TSS) and dye removal which decreased during the illumination time. The effects of different Fe_2_O_3_ NPs based on the varying precursor solution molarities namely F1, F3, F5 and F7 on the wastewater remediation were investigated, and the optimum Fe_2_O_3_ NPs were found to be F1, which exhibited the highest dye removal value of 83% and a chemical oxygen demand (COD) reduction of 88%. Furthermore, the oxidation kinetics of the Procion Blue dye were studied, and the data were well fitted with the second order kinetics. Finally, the thermodynamic parameters including the changes in the Gibbs free energy of activation, entropy of activation and enthalpy of activation for the Procion Blue oxidation with different Fenton's reagent sources under ultraviolet light illustrated that the reaction was non-spontaneous and endothermic.

## Abstract 2

The aim of the present study was to signify the role of the particle size of an iron source in the photo-Fenton system for textile dyeing wastewater oxidation.

## Graphical abstract

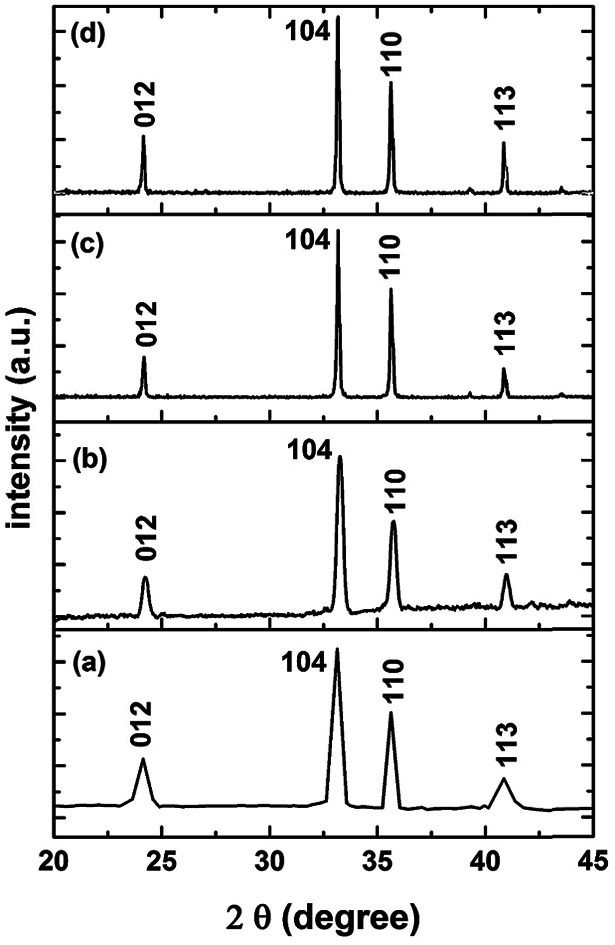

## 1. Introduction

Significant amounts of water are consumed during the dyeing and finishing processes in the textile industry.^1^ Generally, for the production of 1 kg of fabrics, nearly 80 to 150 litres of water is consumed.^2^ Consequently, huge volumes of wastewater effluents containing dyes are discharged, which are rated as one of the most polluting effluents among the industrial sectors. The release of these dyes in the textile effluent discharge is a critical environmental issue because of their high toxic impact, colour and resistance for biological degradation.^1^ Among the several types of the commercial textile dyes produced, reactive dyes represent about 12% of the global production.^3^ Extensive use of these reactive dyes is observed in the textile industry due to the ability of the dyes' reactive groups to form covalent bonds, which help to fix the dyes into the textile fibres. Therefore, the interactions between the textile fibres and the dyes are accelerated, and the total energy consumed for the process is reduced.^4^ Procion MX series dyes are a type of reactive dye that are excellent for direct applications in fabric dying.^2^ About 20% of those dyes are unfixed to the textile and released into the wastewater effluents.^5–7^ Consequently, major environmental deterioration and toxic effects to humans occur. Such effects include cell mutation, skin irritation, eczema and allergies, as well as cacogenic effects.^2^

Presently, great attempts have been made to treat such toxic effluents to substitute conventional techniques.^8–10^ Conventionally, adsorption, flocculation and chlorination were applied for many decades to treat the effluents.^11–13^ However, such techniques are unfavourable since they are non-destructive as they transfer the pollutant from phase to another one without destroying it. Thus, the result is a new kind of pollution that requires secondary treatment.^14,15^ The development of new advanced techniques, called advanced oxidation processes (AOPs), is growing in importance since they are environmentally-friendly processes that produce harmless end products, CO_2_ and H_2_O.^8,16–18^ AOPs are based on the generation of *in situ* ˙OH radicals involving several systems such as chemical oxidation, electro-catalytic oxidation, photochemical reaction and Fenton's reagent. These radicals are the main reason for the organics remediation in wastewater. Fenton's reagent, one of the AOPs, is considered an efficient method for producing a high yield of ˙OH radicals from the reaction between H_2_O_2_ and Fe^2+^ in an acidic wastewater medium.^18–22^ Various Fenton's reagent studies have been suggested in the literature for treating dye-containing effluents. For example, El Haddad *et al.*^23^ treated Reactive Yellow 84 dye using Fenton's reagent; Abou-Gamra^24^ treated Amaranth Red in aqueous effluents, Khan *et al.*^25^ and Liu *et al.*^26^ applied the regent for treating Methylene Blue dye in wastewater.

On the other hand, although Fenton's reagent is the most prevalent AOP for mineralizing several organics, the homogenous Fenton's system has some disadvantages. The most significant issue is the presence of a considerable amount of iron salt in the final treated water sludge that limits the overall system efficiency, as it needs a further treatment. A number of researchers have examined the use of alternative iron source in the Fenton reaction for maximizing the process efficiency using the heterogeneous Fenton's system. Nanosized iron materials are used to replace iron salt in the Fenton's reagent process. For instance, some investigations applied nano-iron compounds for treating different pollutants present in wastewater. Choi and Lee^27^ treated trichloroethylene in water, Prucek *et al.*^28^ removed phenolic compounds, Dutta *et al.*^29^ treated olefins, Nie *et al.*^30^ removed humic acids and Chen *et al.*^31^ treated antibiotics in water. However, according to the authors' knowledge, there is a lack of research on treating Procion Blue MX-7RX in wastewater effluents using Fenton's reagent based on nanosized iron salts.

Many attempts have been made in the last decades to synthesize nanocatalysts.^10,32–35^ According to the literature, NP materials are applied extensively in the field of wastewater treatment. Various methods have been used for manufacturing the nanoparticles, for instance, the wet chemical method,^32^ ionic liquid synthesis,^10^ microwave enhanced techniques,^32^ and electrochemical processes.^36^ In this study, a low-cost, simple sol gel technique was used to synthesize nanoparticles.

Herein, the synthesis of four different Fe_2_O_3_ NPs based on different precursor molarities was explored which directly affected the particle sizes of the investigated Fe_2_O_3_ NPs. Ultraviolet radiation assistance as well as the presence of H_2_O_2_ peroxide was used along with the prepared Fe_2_O_3_ NPs to promote the photo-Fenton reagent catalyst. Different process parameters were examined and compared for the removal of Procion Blue in real textile dyeing wastewater. Moreover, kinetic order and thermodynamic parameters were investigated.

## 2. Materials and methods

### 2.1. Synthesis of Fe_2_O_3_ nanoparticles

The sol–gel route has been used to synthesize Fe_2_O_3_ nanoparticle samples. Reagents of analytical grade were used in the synthesis without additional purification. The used synthetic procedure has been reported elsewhere^37,38^ with some modifications in the used iron oxide precursor concentration. In the typical procedure, both methanol and diethanolamine [HN(CH_2_CH_2_OH)_2_, DEA] were used as the solvent and chelating agent, respectively. The used iron oxide precursor was ferric chloride hexahydrate (FeCl_3_·6H_2_O). The molarity concentration of FeCl_3_·6H_2_O was varied from 0.1 to 0.7 M in order to obtain the four investigated Fe_2_O_3_ nanoparticle samples. For all the synthesized samples, the molarity ratio of DEA to FeCl_3_·6H_2_O was kept at 1 : 1. The desired mass of FeCl_3_·6H_2_O corresponding to the required molarity concentration was dissolved in 20 mL of methanol by stirring at 60 °C for 1 h. Thereafter, DEA was added gradually to the solution and stirred under the same conditions for another 1 h. The obtained homogeneous gel was refluxed at 90 °C in an oven for 6 h. Finally, the investigated powder samples were obtained after calcination at 500 °C for 2 h. All samples were a fine and black powder. The obtained samples are labelled as F1, F3, F5 and F7, referring to the used molarity concentrations of FeCl_3_·6H_2_O of 0.1, 0.3, 0.5 and 0.7%, respectively.

### 2.2. X-ray diffraction characterization

**Fig. 1:**
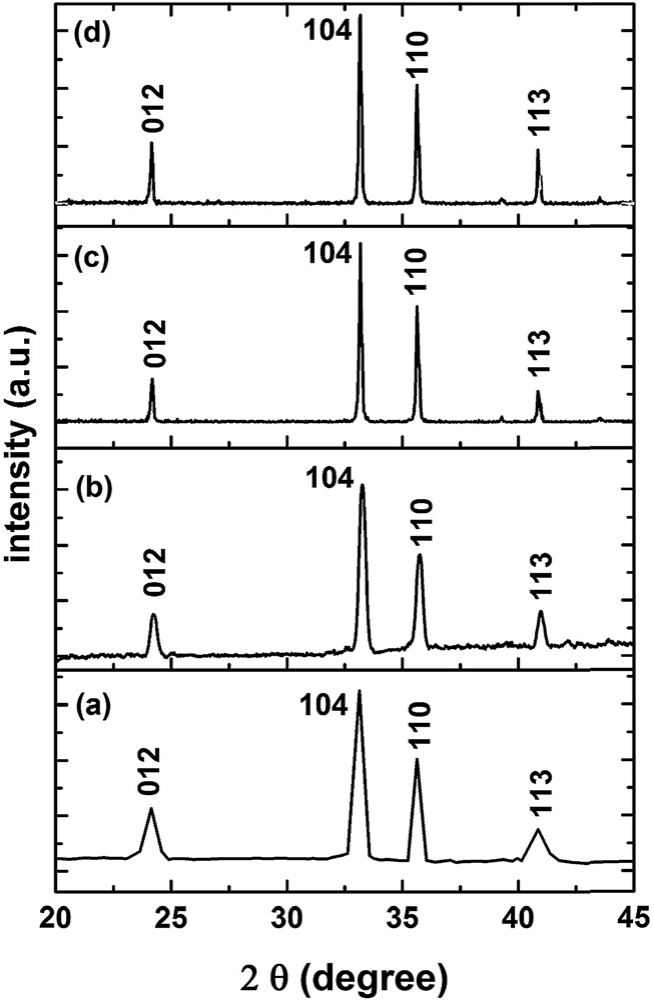
XRD patterns of the synthesized Fe_2_O_3_ samples: (a) F1 sample, (b) F3 sample, (c) F5 sample and (d) F7 sample.

**Fig. 2:**
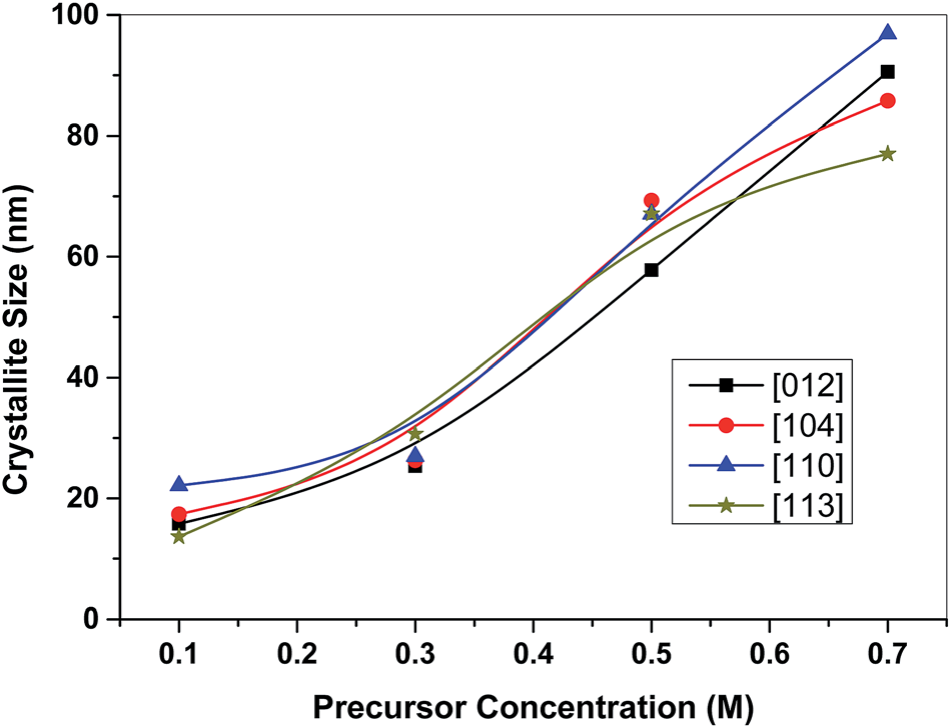
Variation of crystallite size of the investigated α-Fe_2_O_3_ samples with the precursor concentration for different planes of orientation.

An XRPhillips X'pert diffractometer, model MPD3040, was used to examine the crystal structure of the synthesized samples. Fig. 1 shows the X-ray diffraction (XRD) patterns of the investigated samples. The obtained patterns are almost identical in the diffraction line positions and confirmed the formation of a rhombohedral structure of α-Fe_2_O_3_ as the recorded lines of the standard crystallographic data (JCPDS 36-1451).^37,39^ This figure reveals the absence of peaks other than the α-Fe_2_O_3_ for all the investigated samples. The only pronounced difference between the examined samples is the variation in the broadening of the diffraction lines. Specifically, with an increase in the molarity concentration of the precursor, the line broadening decreased which is due to the expected decrease in the crystallite size. The same results have been reported for ZnO and NiO.^29,40,41^ Indeed, the shape, size and phase composition of the synthesized particles using a certain synthetic technique can be controlled by the preparation conditions. Fig. 2 confirms the dependence of the crystallite size of the synthesized samples on the molarity concentration of the precursor as shown for the planes of orientation [012], [104], [110] and [113]. The crystallite sizes (*D*) were calculated using X-ray line broadening using Scherrer's relation^42,43^ for such planes. Fig. 2 reveals the nanosized structure of the formed crystallites for all investigated samples in the selected planes of orientation.

### 2.3. Wastewater sample collection

The reactive dye Procion Blue MX-7RX (reactive blue 161), which is considered to be one of the widely consumed dyes in the textile industry, was selected for treatment from a real textile stream. Procion Blue has a molecular weight of 700 g mol^−1^.^4^ Dye-containing samples were collected from a local dyeing facility in Shebin El-Kom City, Menoufia Governorate, north of Egypt in the period from April to July 2018. The samples were collected after garment washing in order to remove the excess dye after the dyeing process with the addition of inorganic salts for colour enhancement. The dye load in the aqueous solution ranged from 38 to 191 ppm. Hence, the collected aqueous solution contained excess dye molecules, inorganic salts, dust and possibly textile pieces. After collection, the samples were stored in a plastic container and preserved in a refrigerator (4 °C) according to the standard method. The real wastewater samples were analysed and the results are displayed in Table 1.

**Table 1:** Physico-chemical characteristics of real wastewater effluent

Parameter (Unit)	SS (mg L^−1^)	TDS (mg L^−1^)	Salinity (mg L^−1^)	Turbidity (NTU)	pH	DO (mg L^−1^)	COD (mg_COD_ L^−1^)
Value	1210	811	600	36	2.8–3	3.4	596

### 2.4. Photocatalytic methodology

The management of the wastewater containing Procion Blue dye was conducted using a physico-chemical technique. Initially, to ensure the separation of solid particles, the collected aqueous solution was subjected to 24 hours of gravity settling without chemical addition. Subsequently, the supernatant solution was filtered (using a quantitative Whatman 22 μm filter paper), then, the resulting solution was subjected to a photochemical treatment.

**Fig. 3:**
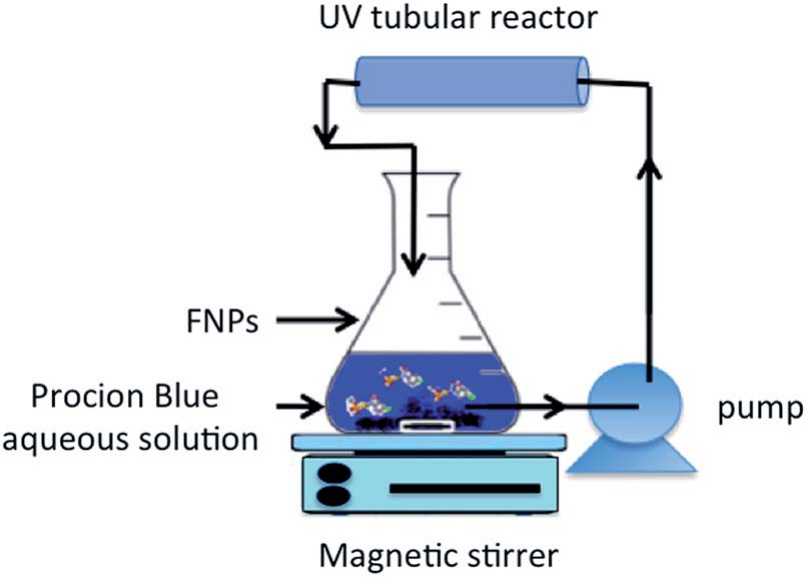
Schematic representation of the photochemical set-up.

Procion Blue photo-oxidation was studied using 500 mL of an aqueous solution in the presence of synthesized iron nano-powder under 60 minutes of UV illumination. The photo-Fenton reagents were initiated by adding various doses of hydrogen peroxide (30%) into the prepared nanoparticles. The pH of the solution was adjusted if needed by adding sulphuric acid and/or sodium hydroxide. Before the solution was submitted to UV illumination the nanoparticles were well dispersed in the solution using sonication. Thereafter, the dye solution with the Fenton's reagent was subjected to UV illumination through a tubular reactor (see Fig. 3). The reactor consisted of a UV-A, 12 W lamp, enclosed in a transparent silica glass tube and covered in a stainless steel jacket. The reaction mixture was circulated in the tubular reactor using a peristaltic dosing pump. Then, within a specific time interval, the treated solution was filtered using a micro filter then subjected to a UV-visible spectrophotometer.

### 2.5. Analytical determinations

The textile wastewater substrate concentration was determined by evaluating its colour removal using a UV-visible spectrophotometer (Unico UV-2100 spectrophotometer, USA) at a maximum absorbance peak of 570 nm. In addition, the chemical oxygen demand (COD) was monitored following the standard procedures of sample digestion.^44^ The total suspended solids (TSS) of wastewater was examined following the standard methods^44^ and the turbidity using an ICM turbidimeter (USA). However, dissolved oxygen (DO) was examined *via* an ICM instrument, USA. The salinity of the wastewater was evaluated by a portable Cole-Parmer EC300 (USA) device. The extent of the pH was determined by a digital pH-meter (AD1030, Adwa Instruments, Hungary).

## 3. Results and discussion

### 3.1. Effectiveness of different oxidation systems

**Fig. 4:**
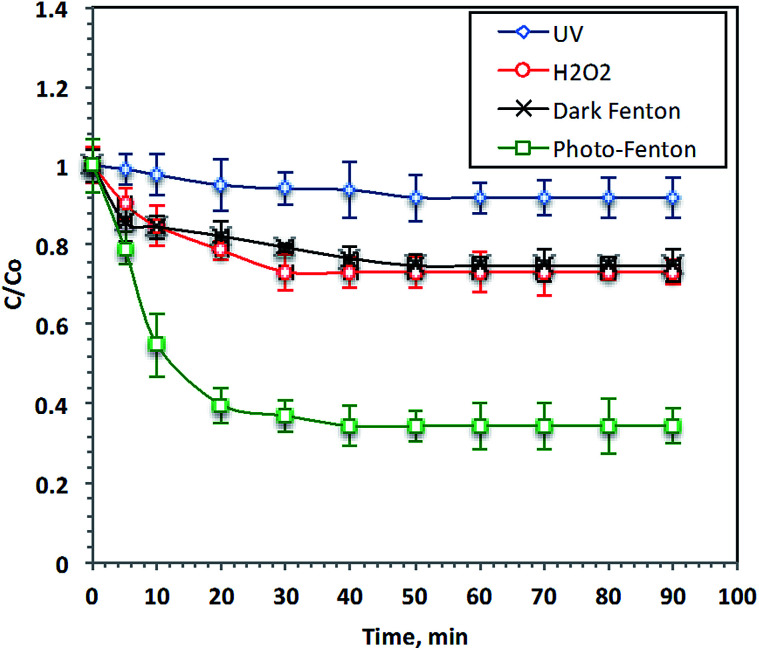
Effect of different oxidation systems on Procion Blue MX-7RX contained wastewater.

The effects of different advanced oxidation processes (AOPs) on the oxidation of Procion Blue wastewater were investigated and their performances are illustrated in Fig. 4. UV illumination, H_2_O_2_, dark Fenton and photo-Fenton were used for 38 ppm-Procion Blue removal. The Fenton's reagent doses were: [F7] = 40 mg L^−1^, [H_2_O_2_] = 800 mg L^−1^ at the starting original pH of the real wastewater (2.8) without further adjustment and [H_2_O_2_] = 800 mg L^−1^ for the hydrogen peroxide oxidation system. The reaction time required to reach the steady state of all oxidation systems was examined, as shown in Fig. 4. Examination of the data reveals that UV-photolysis alone only achieved 8% of the dye removal after 90 minutes of irradiation time. However, the addition of H_2_O_2_ to the wastewater under UV illumination increased the Procion Blue removal to 28%. However, the addition of Fenton's reagent without UV irradiation achieved only 25% removal. The Procion Blue dye-containing wastewater removal increased to 66% when the Fenton's reagent was added under UV radiation. Clearly, the reaction between the F7 and H_2_O_2_ produces the ˙OH radicals that play an important role in the dye oxidation. As stated in the literature,^4^ Procion Blue MX-7RX dye molecules consist of aromatic rings. The ˙OH radicals produced during the reaction with Fenton's reagent are sufficient for attacking those aromatic rings and then opening them ultimately transferring them into harmless end products (CO_2_ and H_2_O). Additionally, it is clear that the exposure to UV illumination in conjunction with the Fenton's reagent achieves a pronounced removal effect. This can be illustrated by the additional reaction intermediates, the ˙OH radicals, produced by the use of UV radiation. Thus, the pollutant removal was enhanced. The results are in accordance with the previous observation of ref. 45 in the degradation of Orange II and ref. 46 for treating several textile dyes using the photo-Fenton reagent.

### 3.2. Effect of initial dye load

**Fig. 5:**
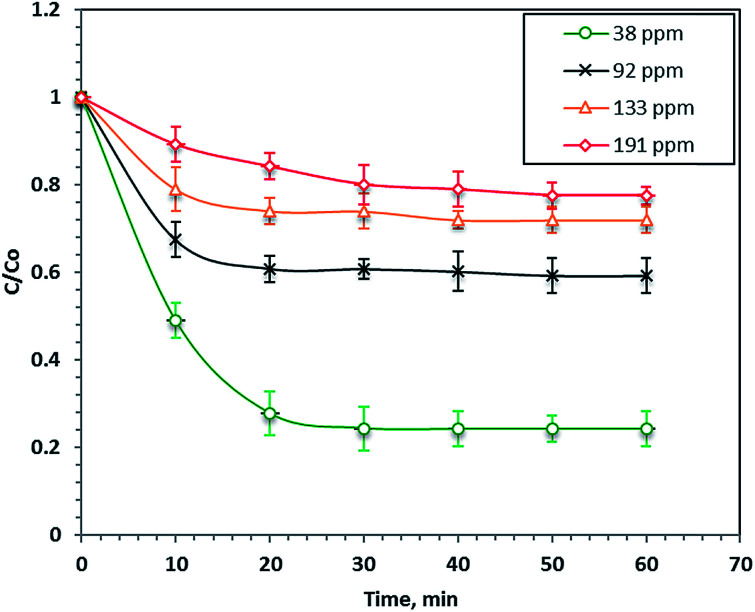
Effect of different Procion Blue MX-7RX loads on the photo-Fenton oxidation system.

The effect of the load of the initial dye concentration in the real wastewater samples on its Fenton's photo-catalytic oxidation (40 mg L^−1^ nanoparticles and 800 mg L^−1^ H_2_O_2_ at pH 2.8) is illustrated in Fig. 5. The results in Fig. 5 demonstrate that the reaction rate after 60 minutes of UV illumination increased with a decreasing initial dye load and the percentage removals were 76, 41, 28 and 23% for the initial dye concentrations of 38, 92, 133 and 191 ppm, respectively. This phenomenon of increasing the dye removal rate with the initial dye concentration can be attributed to the increase in the colour of the wastewater that causes a shadowing effect that prevents the UV illumination from penetrating the aqueous solution. Hence, the ˙OH radicals decreased and thus, the overall reaction rate decreased. Furthermore, the COD of the dye decreased the final treated wastewater to 88%. Najjar *et al.*^47^ previously observed an increasing photo-oxidation rate with decreasing initial organic loads.

### 3.3. Effect of iron concentration

**Fig. 6:**
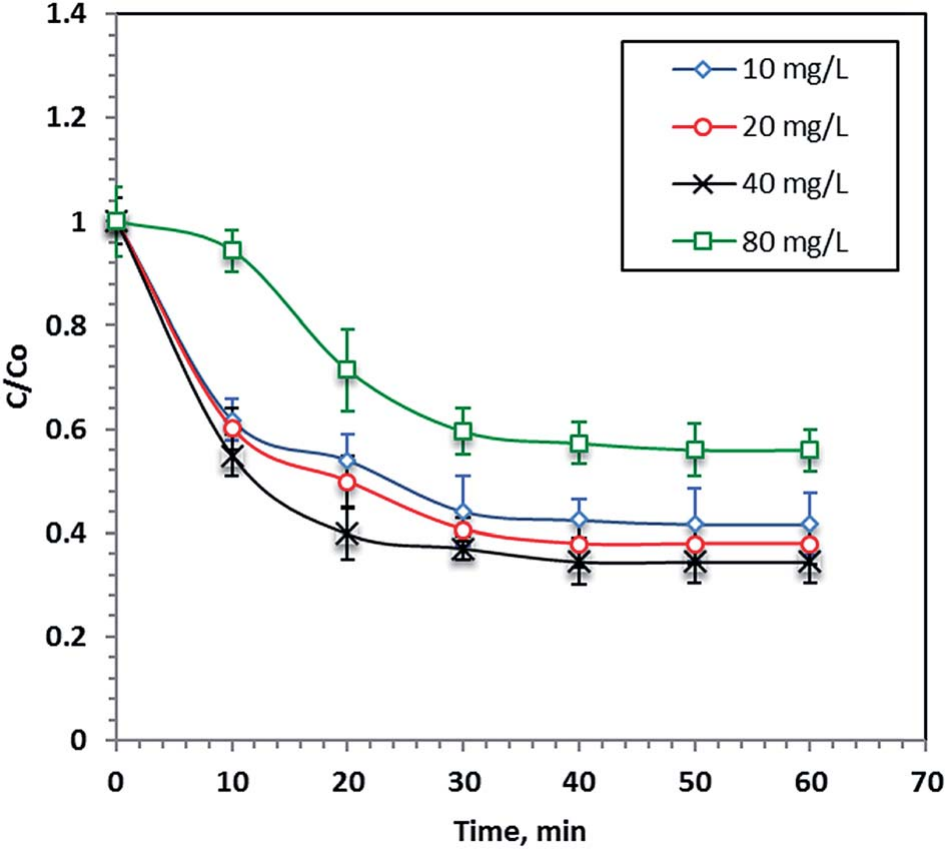
Effect of F7 concentration on the photo-Fenton oxidation system.

Different F7 concentrations (10–80 mg L^−1^) were undertaken at a constant hydrogen peroxide concentration (800 mg L^−1^) and a pH of 2.8 to investigate the role of nano-iron concentration on the photo-Fenton process during 60 minutes of reaction time. The results in Fig. 6 show that an increasing F7 concentration increases the dye removal rate and the optimum F7 concentration was 40 mg L^−1^, which results in 67% of dye removal within 60 minutes of reaction time. However, increasing the iron concentration more than this optimal value reduces the process performance. This finding can be illustrated by the fact that the presence of excess iron salt results in the production of more iron ion species rather than the more useful ˙OH radicals, which were responsible for the dye removal. Furthermore, the excess iron species resulted in a turbid solution and thus the UV light penetration was reduced. Hence, the overdosing of nanoparticles in the solution inhibits the overall reaction rate.^48^

### 3.4. Effect of H_2_O_2_ concentration

**Fig. 7:**
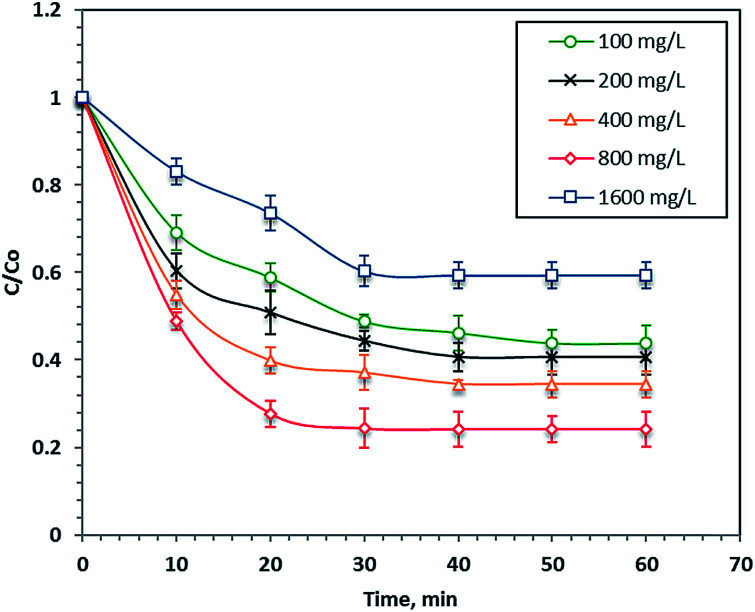
Effect of H_2_O_2_ concentration on the photo-Fenton oxidation system.

Fig. 7 illustrates the effect of various doses of hydrogen peroxide ranging from 100 to 1600 mg L^−1^ where all other parameters were kept constant (40 mg L^−1^ of nanoparticles at pH 2.8). The results in Fig. 7 display a significant enhancement in the removal rate with an increase of the hydrogen peroxide reagent after 60 minutes of reaction time. This increment in the reaction rate was clearly associated with the ˙OH radicals produced since the hydrogen peroxide concentration was a key factor that significantly influenced the generation of the radicals. Conversely, when the H_2_O_2_ concentration exceeded 800 mg L^−1^, the optimal value, the overall reaction yield decreased as the excess hydrogen peroxide reacted with the ˙OH radicals rather than producing them. Thus, the number of available ˙OH radicals in the reaction medium that were responsible for degrading the organic molecules decreased. Furthermore, perhydroxyl radicals, HO_2_˙, which are weak and insignificant radicals for oxidation, were produced instead of ˙OH radicals. Therefore, the overdose H_2_O_2_ concentration acted as a so-called scavenging effect.^20,49–51^

### 3.5. Effect of pH

**Fig. 8:**
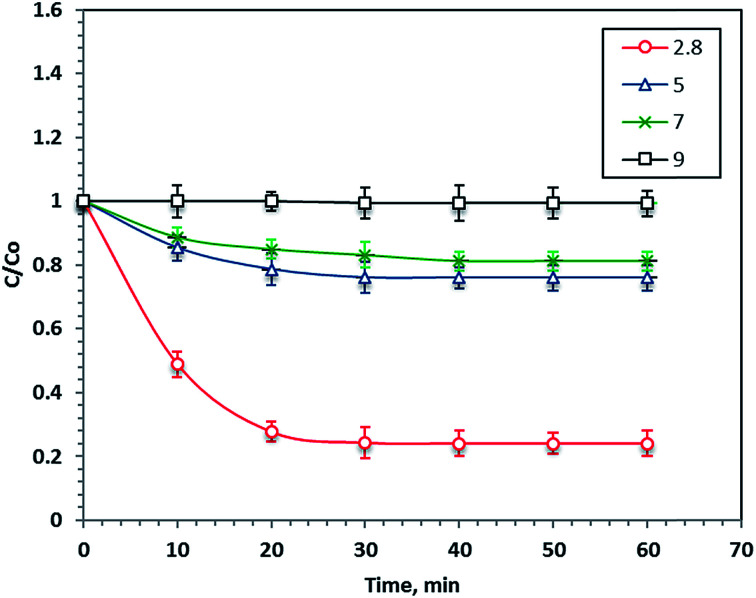
Effect of pH on the photo-Fenton oxidation system.

pH is considered an important parameter in the Fenton's reaction since it significantly affects the ˙OH production. The optimum pH value influences the decomposition of H_2_O_2_ and the hydrolytic speciation of iron particles. To investigate the pH effect for Procion Blue removal, 40 mg L^−1^ of F7 and 800 mg L^−1^ of H_2_O_2_ were added to different wastewater samples at various starting pH values ranging from 2.8 to 9.0 under 60 minutes of UV illumination. Noticeably, the Procion Blue removal rate was highly dependent on the initial pH of the solution, as shown in Fig. 8. Decreasing the pH value increased the reaction removal rate to 76%. However, a neutral pH decreased the reaction rate to 18%. Also, alkaline wastewater resulted in 1% less removal. This is illustrated by the fact that the optimal pH (2.8) enhanced the ˙OH production to the maximum value. Additionally, the reaction media at the acidic pH conditions including the organometallic complex where more H_2_O_2_ was regenerated, thus the reaction rate was increased.^52^ However, increasing the pH value was unfavorable since undesired radicals were produced rather than ˙OH radicals, which reduced the reaction rate. This finding of the sensitivity of the Fenton's reaction to working at a pH of 3.0 was previously investigated in the literature.^15,53^ However, it should be mentioned that the desired acidic pH for maximal removal results in a low pH in the treated effluent. Thus, that the acidic pH conditions required for treatment appear in the final effluent is one of the most important issues to be overcome in the Fenton's reaction for reaching a commercial application. Obviously, further research work should be conducted on this aspect.

### 3.6. Effect of different Fe molarities

**Fig. 9:**
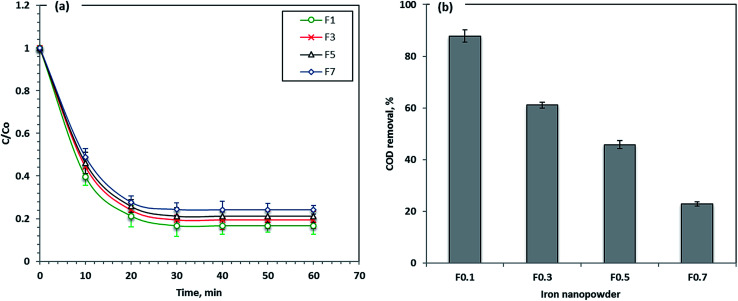
Effect of different photo-Fenton iron salts on Procion Blue removal.

Four different prepared nanoparticle samples labeled as F1, F3, F5 and F7 were applied to check their capabilities for Procion Blue removal. The results in Fig. 9 revealed that the removal efficiency of the materials used in Procion Blue oxidation was in the order of F1 > F3 > F5 > F7. F1 was the most efficient photooxidant in the Fenton's reaction for this dye with a removal rate that reached 83% compared to 76% for F7. Furthermore, the COD after treatment was recorded for the different treated samples and it was confirmed that the maximum COD removal reached 88% for F1 followed by 61, 46 and 22% for F3, F5 and F7, respectively. Additionally, according to our preliminary investigation, all the nanomaterials applied in this study could be used successively, for up to 5 cycles, with a removal efficiency reduction of 30% compared to the fresh ones (the data of this study is not included). Thus, this confirmed the sustainability of such material.

Here it worth mentioning that the obtained average crystallite sizes for the investigated samples were found to be 17.2 nm, 27.3 nm, 65.3 nm and 87.6 nm for F1, F3, F5 and F7 samples, respectively, as obtained using Fig. 2. This suggests that the F1 sample has the highest surface area with respect to the other samples that could increase its photocatalytic activity in comparison to the other samples. So, the reduction in the surface area between F1 and F7 can be attributed to the coalescence of micropores to form larger pores at a higher precursor molarity.^40^ Thus, F1 is a suitable photocatalyst for Procion Blue removal.

### 3.7. Effect of temperature

**Fig. 10:**
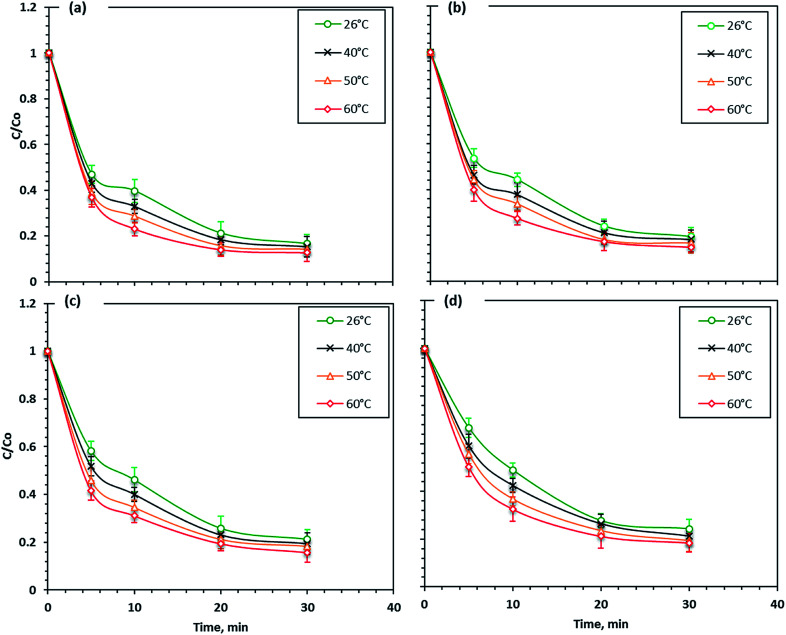
Temperature effect on different Fenton's systems (a) F1, (b) F3, (c) F5, (d) F7.

The wastewater effluent temperature was considered a critical factor that affects the photo-Fenton reactions. Various solution temperatures were checked to determine their effect on the Fenton reaction; the reaction was carried out at 26, 40, 50 and 60 °C. As shown in Fig. 10, the Fenton's reaction rate using different iron nano-powder samples was slightly increased with an increasing temperature. The maximum dye removal efficiency increased from 76 to 82% with the temperature increase from 26 to 60 °C when F7 nanoparticles were used, which illustrates that the Fenton's reaction on the Procion Blue dye photo-oxidation was endothermic. Also, a similar trend was seen in the reaction rate for the F5, F3 and F1 nanoparticles. The dye removal increased with increasing temperature to reach 84, 85 and 87% for F5, F3 and F1, respectively.

Thus, compared to the other operational parameters, the temperature effect on the Fenton's reagent had a small positive effect on the reaction enhancement. Therefore, the enhancement in the removal rate with the temperature increase was probably because at higher temperatures the collision frequency of molecules in the solution increased.^47^ This result means that even the increase in temperature enhanced the reaction kinetics and at higher temperatures hydrogen peroxide may have decomposed to O_2_ and H_2_O^54^ and thus, ˙OH radicals were reduced. According to the literature,^55,56^ the efficient working temperature for the Fenton's reaction is recommended to be 17 and 38 °C.

### 3.8. Determination of oxidation kinetics

The oxidation of Procion Blue kinetics by Fenton's reagent were estimated at various temperatures changing from 26 to 60 °C. Then, the data were regressed for zero-, first- and second-order of kinetics and the values were plotted (plots are not shown) and the slope and intercept of these plots were used to determine the kinetic rate constants (*k*_0_, *k*_1_ and *k*_2_). The reaction kinetic constants *k*_0_, *k*_1_ and *k*_2_ and the reaction half-life (*t*_1/2_) at different temperatures are listed in Table 2. It is observed that higher values of *R*^2^ for the second order kinetics for all Fenton's reagent systems applied. Hence, the Procion Blue oxidation followed second order reaction kinetics. Furthermore, it is noted that with the temperature increase, the second order rate constant (*k*_2_) increased, however, the reaction half-time (*t*_1/2_) increased. This investigation is in agreement with that previously stated in the literature for treating different dyes using Fenton's reagent.^57–59^

**Table 2:** Kinetic parameters of Procion Blue oxidation by different photo-Fenton's systems

Fenton's system	*T*, K	Zero-order reaction kinetics			First-order reaction kinetics			Second-order reaction kinetics		
		*k*_0_, min^−1^	*R* ^2^	*t*_1/2_, min	*k*_2_, L mg^−1^ min^−1^	*R* ^2^	*t*_1/2_, min	*k*_2_, L mg^−1^ min^−1^	*R* ^2^	*t*_1/2_, min
F1/H_2_O_2_/UV	305	0.79	0.72	21.28	0.79	0.91	0.88	0.005	0.99	5.9
	313	0.78	0.67	21.47	0.057	0.88	12.15	0.0055	0.98	5.4
	323	0.78	0.62	21.57	0.06	0.84	11.55	0.0062	0.95	4.8
	333	0.78	0.59	21.52	0.063	0.81	11.00	0.007	0.95	4.3
F3/H_2_O_2_/UV	305	0.79	0.77	21.25	0.053	0.89	13.10	0.0042	0.99	7.1
	313	0.76	0.69	22.08	0.053	0.88	13.07	0.0045	0.97	6.6
	323	0.78	0.67	21.52	0.056	0.85	12.30	0.0051	0.95	5.8
	333	0.77	0.62	21.80	0.057	0.83	12.16	0.0057	0.97	5.2
F5/H_2_O_2_/UV	305	0.79	0.8	21.24	0.0503	0.93	13.78	0.0038	0.99	7.8
	313	0.78	0.74	21.52	0.052	0.9	13.33	0.0042	0.98	7.1
	323	0.76	0.67	22.08	0.052	0.85	13.28	0.0044	0.97	6.8
	333	0.77	0.64	21.80	0.056	0.85	12.39	0.0053	0.98	5.6
F7/H_2_O_2_/UV	305	0.79	0.83	21.25	0.047	0.93	14.53	0.0033	0.97	9.0
	313	0.78	0.78	21.52	0.048	0.92	14.44	0.0037	0.99	8.1
	323	0.79	0.74	21.25	0.052	0.9	13.25	0.0042	0.98	7.1
	333	0.77	0.68	21.80	0.053	0.86	13.10	0.0045	0.97	6.6

### 3.9. Oxidation thermodynamics

To further understand the oxidation process associated with Fenton's oxidation of Procion Blue, thermodynamic parameters were explored. In order to address these thermodynamic parameters, the Procion Blue dye oxidation was evaluated by the second order kinetic rate constant, activation enthalpy (Δ*H*′′), the activation entropy (Δ*S*′′) and Gibbs free energy of activation (Δ*G*′′) using the following eqn (1)–(3). In addition, the energy of activation was obtained from the slope of (−*E*_a_/*R*) of the plot in Fig. 11.1
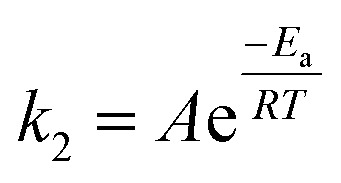
where: *k*_2_ is the second order kinetic rate constant (L mg^−1^ min^−1^), *E*_a_ is the activation energy (kJ mol^−1^), *R* is the gas constant (8.314 J mol^−1^ K^−1^), *T* is the temperature (K) and *A* is the Arrhenius factor.^54^ However, Eyring's equation^60,61^ was applied to calculate the Gibbs free energy of activation (Δ*G*′′) using the activation energy and the rate constants, *k*_2_ values as eqn (2):2
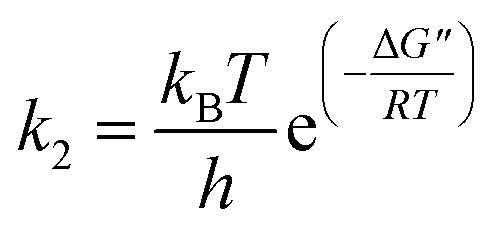
where *k*_B_ is the Boltzmann constant (1.3805 × 10^−23^ J K^−1^), and *h* is Planck's constant (6.6256 × 10^−34^ J s). Additionally, the activation enthalpy (Δ*H*′′) and the entropy of activation (Δ*S*′′) were estimated from eqn (3) and (4).^62^

3 Δ*H*′′ = *E*_a_ − *RT*

4
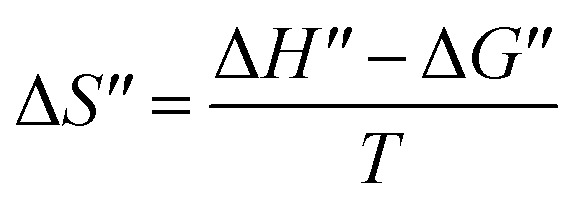


**Fig. 11:**
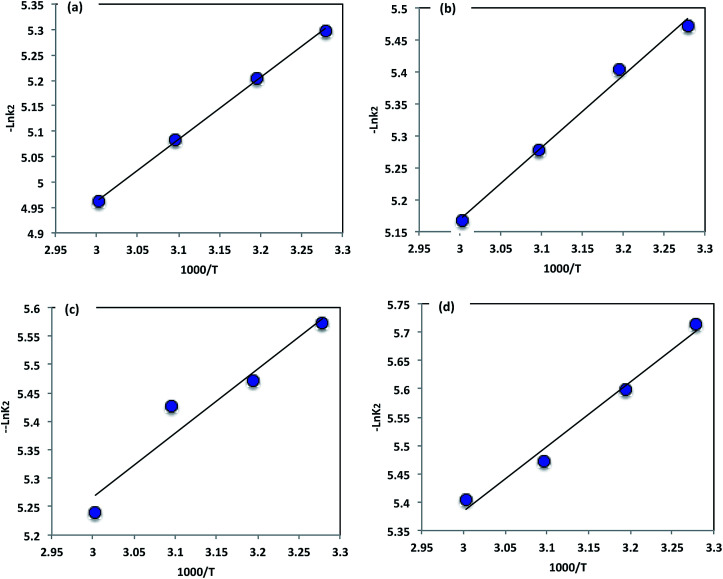
Arrhenius plot of the pseudo second-order kinetic constants: (a) F1/H_2_O_2_/UV, (b) F3/H_2_O_2_/UV, (c) F5/H_2_O_2_/UV, (d) F7/H_2_O_2_/UV.

The calculated thermodynamic parameters for the oxidation of Procion Blue using different iron nanoparticles as a source of Fenton's reagent are tabulated in Table 3. The positive value of Δ*H*′′ indicates the reaction is endothermic, and the positive values of the Gibbs free energy of activation (Δ*G*′′) indicate the process was non-spontaneous. Further, the negative value of the entropy of activation (Δ*S*′′) confirms the process is non-spontaneous. The results show a reduction in the degree of freedom of the dye species and suggest a strong reaction between the hydroxyl radicals and the dye molecules.

**Table 3:** Thermodynamic parameters for Procion Blue oxidation by different Fenton systems

Fenton's system	Temperature (K)	ln *k*_2_	*E*_a_ (kJ mol^−1^)	Δ*G*′′ (kJ mol^−1^)	Δ*H*′′ (kJ mol^−1^)	Δ*S*′′ (J mol^−1^ K^−1^)
F1/H_2_O_2_/UV	305	−5.29	10.15	88.63	6.84	−268.18
	313	−5.20		90.85	6.77	−268.61
	323	−5.08		93.50	6.69	−268.76
	333	−4.96		96.17	6.60	−268.96
F3/H_2_O_2_/UV	305	−5.47	9.37	88.19	7.61	−264.20
	313	−5.40		90.32	7.54	−264.47
	323	−5.28		92.97	7.46	−264.74
	333	−5.17		95.59	7.38	−264.93
F5/H_2_O_2_/UV	305	−5.57	9.4	88.89	6.83	−269.03
	313	−5.47		91.02	6.77	−269.19
	323	−5.43		93.89	6.68	−269.99
	333	−5.24		96.37	6.60	−269.57
F7/H_2_O_2_/UV	305	−5.72	9.5	89.24	69.41	−269.85
	313	−5.59		91.35	68.74	−269.90
	323	−5.47		94.02	67.91	−270.04
	333	−5.40		96.82	67.08	−270.61

However, it is interesting to note that the thermodynamic values show an increase in the Gibbs free energy of activation and enthalpy of activation when comparing F1 through F7, as shown in Table 3. The increase in the positive values of enthalpy means more energy was gained to complete the endothermic reaction^62^ for F7 than F1. Further, the enthalpy of activation of the Fenton reaction using different nanoparticles was in the order of F7 > F5 > F3 > F1. Hence, the reaction was favoured at a low enthalpy value when F1 was applied as the source of Fenton's reagent. This confirmed the differences in the oxidation effect of those materials. This agrees with the previous finding of Argun and Karatas^63^ who reported the Fenton's reaction as endothermic.

## 4. Conclusion

Different Fe_2_O_3_ nanocrystalline powders can be readily synthesized *via* a sol gel technique with different surface areas. In this work searching for the optimal operating parameters for the photo-oxidation of Procion Blue MX-7RX, the pH value was found to be the most affecting factor among the Fenton's reagent parameters changed in the reaction medium. The optimal pH value found was 2.8, which is the natural pH of the real dye-containing wastewater and the dye removal reached 76% at this value. Furthermore, H_2_O_2_ and nanopowder iron concentrations were recorded at 800 and 40 mg L^−1^, respectively. Moreover, different nanoparticles based on various molarities were synthesized which affected the size and led to a variation in the properties of the prepared nanopowder and thus affected the dye removal. However, a determination of the most efficient particle size should be accomplished by an economic study based on both the yield and the cost of each prepared nanoparticle as well as the possible number of cycles of the reaction. The potential of the dye removal followed a second order kinetic rate. The dependence on temperature of the oxidation of Procion Blue was investigated and the thermodynamic parameters were calculated and indicated that the reaction is endothermic and non-spontaneous. This investigation provided the concept that Fenton's reagent can be retained as a photocatalyst for Procion Blue removal in textile effluent wastewater.

## Conflicts of interest

There are no conflicts to declare.

## Supplementary Material
